# Pharmacogenetics to Avoid Adverse Drug Reactions

**DOI:** 10.3390/jpm12020159

**Published:** 2022-01-26

**Authors:** Luis A. López-Fernández

**Affiliations:** Servicio de Farmacia, Instituto de Investigación Sanitaria Gregorio Marañón, Hospital General Universitario Gregorio Marañón, 28007 Madrid, Spain; llfernandez@salud.madrid.org

Although a cure is the main goal of a treatment, serious adverse reactions associated with these treatments are a major problem in clinical practice and cost a lot of money for health systems. These adverse reactions limit the success of medicines and can even lead to the death of patients. Identifying the DNA variants associated with adverse drug reactions can help to personalize medicine and sustain health systems. In this way, there are multiple and successful examples of pharmacogenetics to avoid severe adverse drug reactions. Simultaneously, cost-effective studies have confirmed the usefulness of the implementation of pharmacogenetics. This Special Issue focuses on these topics.

In this number, seven original papers and four reviews have spread the knowledge on this critical issue. The pharmacogenetic works presented in this Issue can be classified in three major areas: cardiovascular, cancer, and the nervous system.

Concerning the cardiovascular area, three original research papers and a review are presented. Angulo-Aguado et al., carried out a pharmacogenetic study of *CYP2C19* in acute coronary syndrome patients of Colombian origin that reveals new polymorphisms potentially related to clopidogrel therapy [1]. They established a pharmacogenetic profile of *CYP2C19* in the Colombian population, and identified a new potentially pathogenic mutation (p.L15H) and several intronic variants. Functional and larger studies are necessary to apply these findings.

Zubiaur et al. studied 60 variants in 15 genes in 156 healthy volunteers enrolled in atorvastatin bioequivalence clinical trials [2]. Genetic analyses showed that variants in *SLCO1B1, CYP3A5, SLC22A1*, and *UGT2B7* are associated with pharmacokinetic variability. This fact suggests that these changes can also affect adverse reactions to this drug.

Statins are used to reduce the risk of adverse cardiovascular events. Solomon et al. analyzed the high-level epistatic risk for on-statin adverse cardiovascular events using genome wide association studies and including up to 5890 individuals [3]. This large study is essential for the progress of this research line. Although the replication of these findings in other populations is desirable, this study opens new ways and will be essential in the future of statins.

In recent years, the biomarkers to avoid adverse reactions in cardiology have been identified and clinical guidelines with recommendations published. In this Issue, a deep and complete review on this subject is presented. The comparison of mainly the Clinical Pharmacogenetics Implementation Consortium and Dutch Pharmacogenetics Working group recommendations is presented and commented on, trying to answer the question: are we ready for implementation? [4]. Thus, drugs, such as acenocoumarol, atorvastatin, clopidogrel, flecainide, metoprolol, phenprocoumon, propafenone, simvastatin, and warfarin, are included in those with recommendations by any of these consortia.

Finally, in the field of cardiovascular diseases, Raymond et al. presented a systematic review about the pharmacogenetics of direct oral anticoagulants [5]. In this work, direct oral anticoagulants, such as dabigatran, riaroxaban, apixaban, edoxaban, and betrixaban, are reviewed, and the association between polymorphism and the exposure variation to them is presented. Thus, the DNA variants in *CES1, ABCB1, CYP3A4*, and *CYP3A5* are identified as relevant in the pharmacogenetics of direct anticoagulants. 

Another field that is well represented in this Issue is cancer pharmacogenetics. Fluoropyrimidines are the base of the treatment in many solid tumors. However, a high proportion of patients receiving these drugs experience a severe adverse reaction that can even be fatal. Fluoropyrimidines exert their anti-oncogenic activity by inhibiting *the thymidylate synthase* gene (*TYMS*). A polymorphism in the enhancer region of *TYMS* has been associated with its transcription rate and subsequently with the efficacy and toxicity of these drugs. However, the results are inconclusive. Schaerer et al., after the sequencing of this region in more than 600 patients, suggested a new nomenclature and demonstrated its usefulness, associating the number of upstream stimulatory factor (USF1)-binding sites with 5-fluorouracil-induced gastrointestinal toxicity [6].

In line with this, Simoes et al. suggested that chemotherapy-induced toxicity in colorectal cancer is likely complex and multigenic [7]. For this reason, they summarized the pharmacogenomic data on colorectal cancer, including candidate gene approaches, genome wide association studies, and next-generation sequencing studies. They also reviewed functional studies, mainly for *DPYD* variants and fluoropyrimidine-induced toxicity, and cost-effectiveness analysis for the clinical implementation of pharmacogenetic tests in colorectal cancer pharmacogenetics.

Drugs used in the nervous system can potentially induce adverse reactions. Since the use of these types of drugs is increasing, the identification of the pharmacogenetic biomarkers to prevent adverse reactions is necessary.

Phenytoin is an anti-epileptic drug that causes cutaneous adverse reactions. Two works are presented in this Issue on antiepileptic drugs. Ahmed et al., reviewed the role of DNA variants in HLA and cytochrome P450 genes in the risk of aromatic antiepileptic-induced severe cutaneous adverse reactions [8]. They summarized the knowledge on HLA, *CYP2C9*, and *CYP2C19,* and its role in severe cutaneous adverse reactions, as well as drugs inducing this adverse reaction, such as carbamazepine, phenytoin, lamotrigine, or phenobarbital.

Furthermore, Shobana et al. found an association between HLA-B*57:01, HLA-B*55:01, CYP2C9*3, and phenytoin-induced cutaneous adverse drug reactions in the South Indian Tamil population [9] (Shobana John et al.). The identification of these pharmacogenetic biomarkers in this specific population is highly relevant because it contributes to expand the knowledge and application of pharmacogenetics in poorly studied populations, and it can contribute to the worldwide expansion of pharmacogenetic implementation.

Antipsychotics are extended drugs used for mental disorders. Many of these drugs are metabolized by the genes of cytochrome P450, which are highly polymorphic and well known. Carrascal-Laso et al. developed a pharmacogenetic model to personalize antipsychotic treatments [10]. As a result, the mean daily doses of these drugs and polytherapy is reduced in these patients. It leads to less risk of drug-related adverse events and the reduction of treatment costs.

Polymedication is one of the greatest challenges in pharmacogenetics. To know how drugs interact between them and what the role is of our genes in these interactions is crucial, especially in aged populations in which polytherapy is very common. The PharmLines is an initiative to study drug–gene, drug–drug, and drug–drug–gene interactions in (es)citalopram therapy [11]. *CYP2C19* intermediate metabolizers were associated with an increased need of drug switching and dose reduction, and being an intermediate metabolizer for *CYP3A4* increases these effects.

## Data Availability

Not applicable.
